# Community-Based Hip Fracture Rehabilitation Interventions for Older Adults With Cognitive Impairment: A Systematic Review

**DOI:** 10.2196/rehab.5102

**Published:** 2016-01-22

**Authors:** Charlene H Chu, Kathleen Paquin, Martine Puts, Katherine S McGilton, Jessica Babineau, Paula M van Wyk

**Affiliations:** ^1^Department of ResearchToronto Rehabilitation Institute-University Health NetworkToronto, ONCanada; ^2^Lawrence S BloombergFaculty of NursingUniversity of TorontoToronto, ONCanada; ^3^Department of KinesiologyFaculty of Human KineticsUniversity of WindsorWindsor, ONCanada; ^4^Library and Information ServicesToronto Rehabilitation Institute-University Health NetworkToronto, ONCanada

**Keywords:** hip fracture, cognitive impairment, community-based rehabilitation, geriatric rehabilitation, outpatient rehabilitation

## Abstract

**Background:**

A hip fracture in older adulthood can result in function and mobility decline. The consequences are debilitating and place a great burden on patients, caregivers, and the health care system. Although inpatient rehabilitation programs have proven effective, the best practices for community-based rehabilitation required to maintain the gains in function and mobility post hospital discharge are currently unknown.

**Objective:**

The aim of this systematic review is to identify and evaluate the evidence on the effectiveness of community-based rehabilitation post hospital discharge interventions for older adults with cognitive impairment (CI) following a hip fracture, and to identify the physical recovery outcomes and measures used in previous studies.

**Methods:**

The methods outlined in the Cochrane Handbook for Systematic Reviews of Intervention were followed and findings were reported using the Preferred Reporting Items for Systematic Reviews and Meta-Analyses guidelines. The search strategy included a combination of text words and subject headings relating to the concepts of CI, dementia, delirium, cognitive reserve, and hip fractures. For a study to be included in the review, it had to involve participants with CI who underwent hip fracture surgery, and consisted of an outpatient intervention that occurred in the participant’s home or community. Peer-reviewed journal articles were identified by searching various databases. Two independent reviewers screened the titles and abstracts to determine which articles comprising of a rehabilitation intervention within a community setting prior to being included for a full article review. A data extraction form and an evidence and quality checklist were used during the full article data analysis and synthesis. A meta-analysis was not conducted due to heterogeneity of measures and outcomes.

**Results:**

The original search resulted in over 3000 articles. Of those, three studies satisfied the necessary criteria to be included in the systematic review. All studies included inpatient and outpatient physiotherapy, with some including a cognitive component, family education, and a discharge assessment.

**Conclusions:**

The findings from this review suggest that community-based rehabilitation post hospital discharge interventions show promising results towards improving various physical function outcomes, mobility, and activities of daily living for older adults with CI following a hip fracture. This review also demonstrates and discusses the current lack of outpatient rehabilitation interventions targeted towards older adults with CI post-hip fracture. Additionally, several substantive gaps that require attention to move this field forward are highlighted.

## Introduction

After experiencing a hip fracture, older adults are typically admitted into sub-acute or hospital care units to receive rehabilitation [1-3]. However, the presence of cognitive impairment (CI) has been an exclusion criterion for patients to access rehabilitation services [4-6]. This misalignment of care is particularly problematic as one study estimated that dementia and CI have 19% and 42% prevalence among older adults with a hip fracture, respectively [7]. Evidence indicates that approximately two thirds of older adults have severe difficulties walking independently outdoors 6 weeks after discharge from inpatient rehabilitation suggesting severe difficulties in returning to community activities after hip fracture [8]. Consequently, older adults with CI and a hip fracture from the community who are unable to maintain or regain their mobility and functional abilities after discharge from inpatient rehabilitation are frequently admitted into a long-term care home in order to meet their daily care needs [9].

Permanent placement into long-term care accrues a high burden of cost which is expected to reach approximately $2.4 billion in Canada by 2041 [4]. Comparatively, the economic burden resulting from a hip fracture was significantly less for a person who returns to the community and receives nursing, physiotherapy, and occupational therapy in their home [4]. With a greater proportion of older adults with increasing medical complexity [10] and health care systems attempting to contain costs, there is an urgent need for rehabilitation programs in the community to deliver care so that the progress gained from inpatient rehabilitation after discharge is maintained for older adults with CI following hip fracture.

Evidence is beginning to accumulate that rehabilitation offered in post-acute or community settings are beneficial to older adults with CI post hip fracture [11,12]. However, to date, the effectiveness of community-based rehabilitation programs for older adults with CI is poorly understood. It is imperative for decision makers, clinicians, and researchers to know the evidence supporting the effectiveness of outpatient community-based rehabilitation programs following a hip fracture on critical patient outcomes, such as mobility, physical function, activities of daily living (ADLs), and living situation after the program. Identifying the aspects of community-based rehabilitation programs that are specific to older adults with CI is essential to inform future initiatives aimed to prevent decline and institutionalization, as well as restore mobility and function among older adults with CI. The primary aim of this systematic review is to evaluate the evidence on the effectiveness of community-based rehabilitation post hospital discharge interventions for older adults with CI following a hip fracture, and to identify the physical recovery outcomes and measures used in previous studies.

## Methods

### Search Strategy and Selection Criteria

The study protocol has been previously published [13]. This review was based on a systematic, comprehensive search of 12 databases (Medline, Medline In-Process, PubMed, PsychINFO, Embase, CINAHL, AMED, Ageline, The Cochrane Database of Systematic Reviews, Central Register of Controlled Trials, Database of Abstracts of Reviews of Effect, and the Allied Health Evidence databases), from their inception to April 2015. The search strategy included a combination of text words and subject headings relating to the concepts of CI, dementia, delirium, cognitive reserve, and hip fractures. The search was limited to English and French articles due to limited resources to review articles in other languages. The literature search was performed by an experienced information specialist (JB). A study was eligible if it included (1) an intervention with a community-based component aimed at maintaining or improving patient physical recovery outcomes, like function, mobility, and dwelling location; (2) a mean age of 65 years or older for participants; (3) analysis of participants with CI; and (4) participants who suffered a hip fracture. Community-based rehabilitation post-discharge was previously defined to include interventions that were initiated once an individual was discharged home from inpatient rehabilitation for a hip fracture [13]. Our definition needed to be revised to include interventions that began during inpatient care and transitioned into the community. However, this review was designed to focus on the outcomes resulting from community-based components. Study designs could be randomized controlled trials (RCTs), prospective (longitudinal), retrospective (longitudinal), cross-sectional, cohort, and quasi-experimental studies. Multiple research designs were included in order to collect a comprehensive overview of the evidence. Publications were excluded if the rehabilitation program or intervention presented did not include or describe a community or home-based component, did not report results of primary data collection (eg, editorials, commentaries), or if the study was targeted for participants with stroke, Parkinson’s disease, or frontal-temporal dementia, as these diseases have different physiological and behavioral markers.

### Study Selection

The titles and abstracts were first screened by two independent reviewers (CC, PMvW). If one reviewer was uncertain about whether the article fulfilled the inclusion criteria, it was included for full-text review. Two reviewers (CC, KP) independently reviewed full-text studies. All disagreements were resolved by consensus with the research team. Regular team meetings were held to discuss articles, any complications or disagreements that arose, and findings from the studies. If multiple articles were written about the same study, only the article with the most information pertaining to the participants with CI was retained. For any articles that were missing information, corresponding authors were contacted for clarification.

### Data Abstraction and Quality Assessment

Two reviewers (CC, KP) independently extracted data from each of the included studies using a standardized excel sheet developed by the research team. This included information about the: study design, aim, location, sampling method, recruitment period, duration, sources of data collection, sample descriptors (eg, size, age, sex, type of hip fracture, type of CI, pre-fracture living location, and discharge location), interventions (eg, components, setting, duration, assessments and scales used), outcomes, details of statistical analyses, and source of funding. If the study was an RCT, attributes of this design were extracted including randomization, allocation, and blinding methods. To objectively measure the quality of the included studies, two reviewers (CC, KP) independently used the Downs and Black checklist [14]. Any disagreements between the scores were discussed and resolved by the consensus of the research team.

## Results

**Figure 1:**
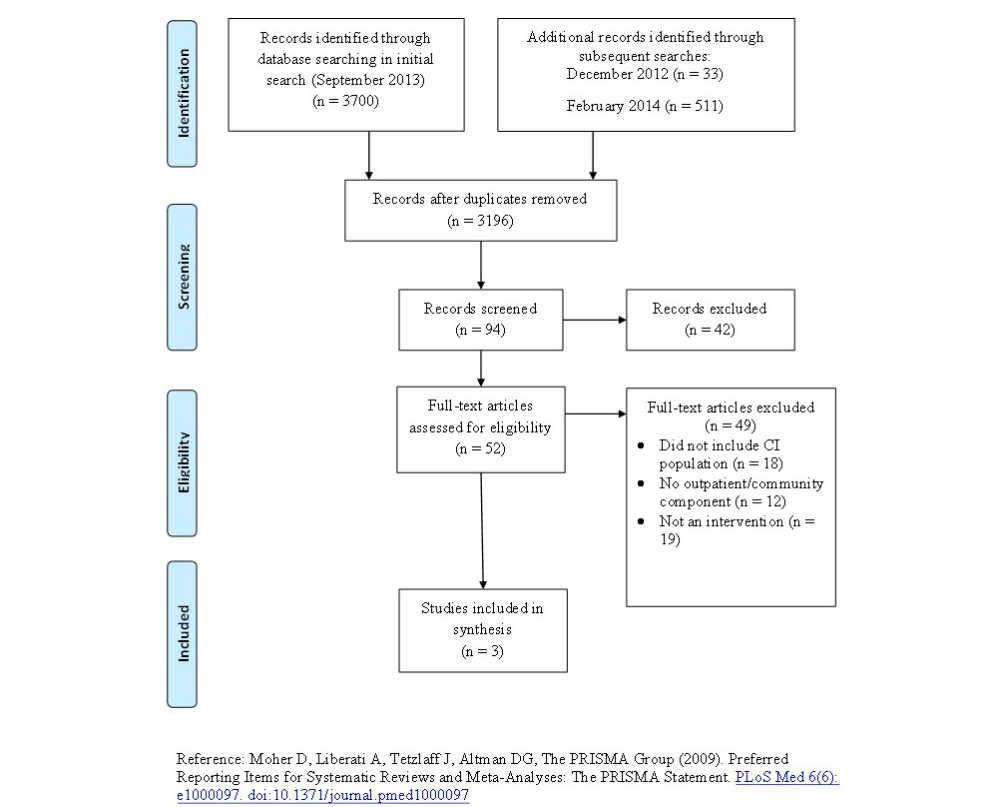
PRISMA diagram of search results.

The initial search in September 2013 yielded 3700 articles. From these results, 1493 duplicates were removed and the remaining 2207 titles and abstracts were screened (Figure 1). A total of 52 full-text articles were deemed eligible. After reviewing the full-text studies, 3 articles were included into the review [15-17]. Although the interventions in these three studies were not specifically designed for only individuals with CI, they did include a sub-analysis for the patient population and thus met our inclusion criteria. The findings from one study was reported in three articles [17-19], but only Shyu et al [17] reported on a subgroup analysis of those with CI with physical recovery outcomes. A meta-analysis was not conducted due to heterogeneity of measures and outcomes. To ensure the review included the most current evidence the search was updated using the same strategy in December 2013, February 2014, and April 2015; no relevant studies were retrieved.

### Characteristics of the Included Studies

Characteristics of the included studies are described (Table 1). All three studies were two-group RCTs with different follow-up periods ranging from 16 weeks post-discharge [16] to 2 years post-discharge [17]. Primary data collection was used in all of the studies; in addition, one study [16] used an administrative database.

**Table 1:** Description of the studies.

Components	Study		
	Huusko et al [15]	Moseley et al [16]	Shyu et al [17]
Study design	RCT	RCT	RCT
Method of randomization	Computer generated	Computer generated	Coin flip
Location	Finland	Australia	Northern Taiwan
Setting	Geriatric ward home	Rehabilitation unit home	General/acute hospital rehabilitation unit home
Recruitment time	October 1994 to December 1998	March 2002 to May 2005	September 2001 to November 2004
Aims	Determining the effect of intensive geriatric rehabilitation after surgery for hip fracture on patients with cognitive impairment	Determining the impact of a higher dose exercise program on mobility after hip fracture compared to usual care	Two-year evaluation of an interdisciplinary intervention program on recovery following hip fracture for older adults with cognitive impairment
Usual care sample size, n	120	80	81
Intervention group sample size, n	123	80	79
Cognitive screening tool	MMSE^a^	SPMSQ^b^	Chinese MMSE
Inclusion criteria	Community dwelling patients with acute hip fractures; ≥65 years; living independently, had been able to walk unaided before the fracture	Surgical fixation for hip fracture admitted to the inpatient rehabilitation; approval to weight bear or partial weight bear; able to tolerate the exercise programs; able to take four plus steps with a forearm support frame and the assistance of one person; no medical contraindications that would limit ability to exercise; living at home or low care residential facility prior to the hip fracture	Age ≥60 years; admitted to hospital for an accidental single‐side hip fracture; receiving hip arthroplasty or internal fixation; able to perform full range of motion (ROM) prior to hip fracture, moderately dependent or better in ADLs before hip fracture^c^; living in northern Taiwan^c^
Exclusion criteria	Pathological fractures, multiple fractures; serious early complications; those receiving calcitonin treatment; terminally ill patients, severe dementia, or other serious problems with communication	High functioning patients who were discharged directly to home; low functioning patients who were discharged to a residential care facility	Severe cognitive impairment (score <10 on the Chinese MMSE); terminally ill
Discharged home	54% community; 46% not reported	Not reported	Not reported
Duration of outpatient component	Unclear	Unclear	3 months

^a^Mini-Mental State Examination

^b^Physical Performance and Mobility Exam

^c^Established to include subjects with the most potential to recover after rehabilitation.

The participant inclusion and exclusion criteria used in each study are outlined in Table 1. In order to be eligible for the trials, inclusion criteria included being 60 years of age of older [15,17], admitted to hospital with a hip fracture [16,17], receiving hip arthroplasty or internal fixation [17], and surgical fixation for hip fracture [16]. The participants’ pre-fracture physical condition was an inclusion criterion in each of the studies.

Sampling methods included convenience [15,17] and stratified [16] sampling. All three studies recruited their participants from hospitals, and the number of participants ranged from 160 [16,17] to the largest sample size which was 243 [15]. The mean age of the study participants ranged from 79-84 years old. The control and the intervention groups were well balanced. The percentages of female participants were 90% [15], 81% [16], and 69.1% [17].

A single measure of CI was used to assess participants in all three studies. In Huusko et al [15] participants were considered to have dementia if they scored less than 23 on the Mini-Mental State Examination (MMSE) which was used to assess patients 10 days after surgery and the randomization process when the patient was “in a clinically stable situation” [15]. In another study, participants were considered “cognitively impaired” [17] using the Chinese MMSE. The third study, Moseley et al [16] used a cut-off score of 3 or more adjusted errors on the Short Portable Mental Status Questionnaire (SPMSQ) which identifies those with no CI and mild CI, but did not further describe the inclusion criteria for CI. Those with SPMSQ scores of 4 or less were excluded. No study used a physician diagnosis of dementia or CI, further no assessment of delirium was considered in the studies.

With respect to describing the participants, only Huusko et al [15] reported the mean level of cognitive function for the control and the intervention groups (MMSE scores were 23 and 20, respectively, *P*<.001), the sample size, and labeled participants with CI as having “dementia.” The two other studies [16,17] did not provide the mean age, percent women, type of hip fracture and treatment, or MMSE score of the participants with CI.

The types of hip fractures and their surgical treatments varied among the studies. Shyu et al [17] enrolled participants with accidental hip fractures who underwent hemiarthroplasty or open internal fixation, the study by Huusko et al [15] comprised of patients who had trochanteric fractures managed with osteosynthesis, and Moseley et al [16] included participants with trochanteric and intracapsular fractures who received either bone screws, compression screws, plates, and hemiarthroplasty as treatment. Two studies had participants who lived in the community [15,16] whereas Shyu et al [17] did not report the pre-fracture living location. The comorbidities of the samples with CI were not described in any of the three studies.

### Quality Assessment

The quality of each study was determined to be 23 [15], 25 [16], and 19 [17], indicating that they are all of “good quality” according to the Downs and Black checklist [14] (Multimedia Appendix 1). Despite the articles being good quality, the Downs and Black checklist identified noteworthy methodological deficits in the three studies: none of the studies attempted to blind study subjects to the intervention, outcome assessors were not blinded, compliance with the intervention was not measured, and randomized intervention assignment was not concealed from both patients and healthcare staff. Furthermore, the studies lacked component analyses of the interventions, descriptive data regarding the participants with CI, reporting on methodological issues (eg, no protocol for missing data), information regarding comorbidities experienced by the participants, and information regarding the intervention acceptability, feasibility, or treatment receipts.

### Interventions

All three of the interventions [15-17] were initiated while the participants were on the inpatient unit. The participants in all three studies received assessments, rehabilitation, home assessments, counseling during inpatient stay and/or discharge planning. The intervention components are listed in Table 2.

Huusko et al [15] referred the intervention group to a geriatric inpatient unit where they would be managed by an interdisciplinary team immediately after randomization whereas the control group was discharged to other hospitals. Their rehabilitation program involved seven intervention components, the highest number of components of all three studies. The seven components included inpatient physical rehabilitation twice a day, cognitive rehabilitation with a psychiatrist four times a week, discharge assessments that involved home assessments and the need for appliances and daily living aids, family education about hip fracture, and registered nurse (RN) and physiotherapists (PTs) weekly meetings to discuss methods of improving rehabilitation. After discharge, participants were provided 10 in-home physiotherapy visits. Information about how long it took to complete the visits or the duration of follow-up was not provided.

The intervention by Shyu et al [17] had six components and began prior to surgery with a geriatric consultation provided by a geriatrician and geriatric nurses. After surgery, the geriatrician provided suggestions to the care team in order to modify or develop a care plan for rehabilitation. The rehabilitation contained six components, including inpatient assessment by a rehabilitation physician, RN and PT, inpatient physical rehabilitation with 2 visits from a PT, daily geriatric nurse visits; comprehensive discharge assessment by a geriatric nurse, and a home assessment prior to discharge. Additionally, Shyu et al [17] included 8 in-home visits from a RN as well as 3 in-home physiotherapy visits in the 3 months following discharge. The control group received routine care which does not include continuity of care, geriatric assessment, an interdisciplinary approach, or in-home visits.

Lastly, Moseley et al [16] provided rehabilitation during inpatient care and continued their exercise regime post-discharge. Their intervention only had two components: high doses of weight-bearing physical rehabilitation that consisted of 1-hour sessions twice a day for 16 weeks, and physiotherapy in the home over 8 visits by a PT after being discharged from the inpatient rehabilitation unit. This was compared to the control group that received usual care with limited weight-bearing exercises. Information about the duration of PTs follow-up was not provided so it was unclear how long patients continued to be seen after discharge.

A geriatric consultation by an interdisciplinary team that typically consisted of a geriatrician, RN, and PT was completed on the inpatient unit in all three studies. In addition to these professionals, a neuropsychologist, social worker, consultant specialist in physical medicine, neurologist, and psychiatrist were involved immediately after admission into the hospital in one study [15]. Geriatric consultants who made suggestions to the surgeon about post-surgery physician orders were also utilized [17].

**Table 2:** Summary of intervention components and outcome measures.

Components		Study		
		Huusko et al [15]	Moseley et al [16]	Shyu et al [17]
**Intervention components**				
	Physical	Physiotherapist visit twice daily; occupational therapy; practice with nurse during day	Weight-bearing exercises twice daily for 60 minutes and walking on the treadmill for 16 weeks	During inpatient stay and 3 months after. inpatient (physiotherapist visits three times daily)
	Cognitive	Psychiatrist up to four times per week	N/A	N/A
At-home physiotherapist		10 visits by a physiotherapist	8 visits by a physiotherapist	3 visits by a physiotherapist
At-home registered nurse		N/A	N/A	4 visits in 1st month, then biweekly until 3rd month
Family education		Family counseling	N/A	N/A
Inpatient assessment		N/A	N/A	Geriatric consultation before and after surgery; nurse and physician visit once a day
Discharge assessment		Discharge plan checked in weekly meetings with the patient and family	N/A	Assessment done by nurse; evaluated (caregiver competence, family resources, family function, patient self-care abilities, and need for community or long-term care services)
At-home assessment		Physiotherapist made home visit before discharge if necessary	N/A	Part of discharge assessment by nurse
Nurse and physiotherapist meetings		Nurse and physiotherapists met weekly to improve rehab	N/A	N/A
Duration of outpatient component		Unclear	Unclear	3 months after discharge
Outcome measures		Length of hospital stay; mortality; place of residence 3 months and 1 year after discharge	Knee extensor strength, and walking speed (primary); PPME, sit to stand, gait aid use, Barthel Index, falls, hospital readmission, pain, EQ5D^a^, balance^b^(secondary)	Hip flexion ration; two items on CBI (walking ability, ADL recovery); falls; mortality; emergency room visits; hospital readmission; institutionalization
Function outcome measures		N/A	Knee extensor strength (primary); PPME, sit to stand, patients rank of strength (secondary); at admission, 4 and 16 weeks; by blinded research assistants	Hip flexion ratio; at 1, 3, 6, 12, 18, 24 months post-discharge; by geriatric nurse
ADL outcome measures		N/A	Barthel ADL scale; PPME; at admission, 4, and 16 weeks; by blinded research assistants	Barthel ADL scale; at 1, 3, 6, 12, 18, 24 months post-discharge; by geriatric nurse
Mobility outcome measures		—	6-minute walking speed test and a self-report measure; at admission, 4, and 16 weeks; by blinded research assistants	One item from Barthel; at 1, 3, 6, 12, 18, 24 months post-discharge; by geriatric nurse

^a^Quality of Life patients rank of strength

^b^Balance: max balance range test, step test, body sway, lateral stability, co-ordinated stability, choice stepping reaction time.

### Outcomes

The outcome measures are outlined in Table 2 and a summary of the results are described in Table 3. Moseley et al [16] used a primary outcome measure of knee extensor strength for which there was no statistically significant between group differences following intervention, or among those with CI. The intervention group had significantly faster sit-to-stand times at both 4 and 16 weeks and performed more steps in the step test at 4 weeks compared to the control group. A post-hoc analysis revealed that those with CI who were allocated to the intervention group had better outcomes than those without both of these factors in physical function outcome measures that included differences in walking speed at 4 (0.20 m/s, *P=*.003) and 16 weeks (0.24 m/s, *P=*.0.15), Physical Performance and Mobility Exam (PPME) at 4 (1.4 units, *P=*.013) and 16 weeks (1.9 units, *P=*.019), body sway at 4 weeks (2.1 cm, *P=*.008), step test at 16 weeks (3.5 s, *P=*.046), max balance range test at 16 weeks (36 mm, *P*=.002), coordinated stability test at 16 weeks (14, *P=*.020), and modified falls efficacy scale at 16 weeks (28, *P=*.009). Having “no or slight pain” (OR=5.3, *P=*.024; difference=0.2, *P=* .034) and being “able to walk unaided or with sticks or crutches” (OR=6.0, *P=* .018) were also significantly improved at 16 weeks for those with CI in the intervention group compared to those in the control group. Huusko et al [15] was not included in this table as their outcome measures were not specifically related to function, ADL, and mobility

**Table 3:** Results of physical function, ADL ability, and mobility outcome measures.

Results	Study	
	Moseley et al [16]	Shyu et al [17]
Physical function	Between group differences of those with CI allocated to intervention group (significant changes in PPME)	No statistically significant results
ADL Ability	Significant improvements for those with CI in the intervention group were reported^a^	Significant improvements for those with CI in the intervention group were reported in both studies^b^
Mobility	Statistically significant findings in those with CI, and found statistically significant improvements for participants with CI in the intervention group compared to those in the control group^c^	Participants with CI in the intervention group were more likely to recover their walking ability compared to the control group^d^

^a^Barthel, *P=*.002; PPME, *P=*.019

^b^ *P=*.001; an increase in Barthel score for those with CI in the control and intervention group 6 months after discharge but it is unclear if this increase was statistically significant.

^c^ *P=*.015

^d^OR=3.49, CI=1.64-7.42, *P=*.001

Shyu et al [17] measured hip flexion ratio and mobility with the walking item on the Chinese Barthel Index (CBI). Results indicated that participants with CI in the intervention group were more likely to recover their walking ability compared to the control group (OR=3.49, CI=1.64-7.42, *P=*.001). However, no statistically significant differences in hip flexion ratio in participants with CI were found [17].

Describing ADLs as a secondary outcome, Moseley et al [16] used the Barthel ADL scale and the PPME whereas Shyu et al [17] only used the Barthel ADL scale. Both studies reported significant improvements for participants with CI in the intervention group (*P=*.002 by Moseley et al [16]; and *P=*.001 by Shyu et al [17]). Moseley et al [16] reported significant findings with both the Barthel (*P=*.002) and PPME (*P=*.019) measures.

Two studies examined whether the intervention impacted participant dwelling location over time and mortality [15,17]. Huusko et al [15] reported that the length of hospital stay for those in the intervention group with mild and moderate dementia was significantly shorter than the control group (*P=*.002 and *P=*.042, respectively). They also found that significantly more participants with mild and moderate dementia from the intervention group were living at home 3 months after discharge (*P=*.009 and *P=*.009, respectively), and continued to live independently 1 year after the operation, though not significantly [15]. In contrast, Shyu et al [17] reported that rates of institutionalization over 2 years were the same between the intervention and control group and that those with CI in the intervention group were most likely to be readmitted into hospital (OR=4.44, CI=1.53-12.89) within the 2-year timeframe. Both studies found no significant differences in mortality between the intervention and control groups. Other outcomes that were evaluated but were non-statistically significant among participants with CI included fall occurrence [16,17], and readmission into the hospital in the intervention group at 16 weeks [16,17].

## Discussion

### Principal Findings

This review demonstrated that there is a current lack of outpatient rehabilitation interventions targeted towards older adults with CI post-hip fracture. Although there has been an increased emphasis on older adults with CI following a hip fracture in rehabilitation interventions, previous studies have primary focused on inpatient settings [11,20]. The three studies that met our inclusion criteria were not designed to meet the specific needs of older adults with CI, which is a similar finding to other literature reviews [21]. Rather, the authors stratified their samples and conducted a subgroup analysis of participants with CI from larger RCTs that aimed to determine the effectiveness of interventions in a geriatric population. The results of this review suggest that community-based rehabilitation post hospital discharge interventions are promising to improve various physical function outcomes, mobility, and ADLs function 1 year post-discharge from the hospital for older adults with CI. Further, there is some evidence to suggest that providing outpatient rehabilitation after discharge from inpatient rehabilitation programs can increase the likelihood of the older adults staying home and avoiding institutionalization for a short (3-month) period of time, but there is insufficient evidence to indicate whether these results were sustained for longer periods of time. Given the increased vulnerability of this patient population, and that CI as a negative prognostic factor for older adults with hip fracture immediately after inpatient discharge [22], these results are potentially significant and warrant more research. Cautious interpretation of the evidence should be exercised as there is a lack of power from the subgroup analysis.

The paucity of studies that deliver an intervention specifically to the population with CI is concerning for a number of reasons. There are approximately 35,000 and rising hip fractures reported annually in Canada [23], and CI is present among almost half of all individuals who experience a hip fracture [7]. Yet, the presence of CI among this patient population has traditionally been a barrier to accessing rehabilitation services [4-6]. The continued exclusion from inpatient rehabilitation of older adults with CI is a concern because they are viable candidates for rehabilitation; evidence shows that older adults with CI can recover from hip fractures and return home when they are provided access to inpatient rehabilitation [24]. However, the current lack of literature evaluating outpatient rehabilitation interventions makes it difficult to determine the feasibility, acceptability, and effectiveness of relevant intervention components for older adults with CI. Our systematic review uncovers the uncertainty of a research topic and provides a baseline of evidence which can contribute to stimulating more robust research [25].

Through conducting this review, several critical insights were gained regarding the design and implementation of outpatient rehabilitation interventions. There was a consensus between the three studies that the outpatient rehabilitation interventions should begin early in the care trajectory while the participants are still receiving inpatient care, and should include discharge planning. Although it was unclear from the studies when exactly the discharge planning began, there is evidence that early initiation of discharge planning improves the continuity of care from an inpatient hospital setting into the community [26]. Maintaining continuity of care is a crucial aspect of geriatric care because older adults recovering from a hip fracture are most at risk during transitions, and inconsistencies in care can negatively impact patients’ ability to maintain the progress they made in inpatient rehabilitation [27].

The other acquired insight is that an interdisciplinary team approach was a shared commonality of the interventions. Physiotherapy visits were included in all of the interventions; unfortunately, the authors poorly described the details of the physiotherapy component. The lack of information regarding the physiotherapy component of the interventions is concerning as there has been an increased emphasis on post-operative physiotherapy and occupational therapy [21]. We have defined this ambiguity as the “black box of physiotherapy”. In addition, because there is no standard evidence-based care practice for this particular population in the community, it is challenging to determine the most appropriate person to deliver the therapy, in the suitable dose, frequency, and intensity, as well as identify outcome measures that are responsive and sensitive to change over time in order to compare and analyze these component characteristics. In addition to physical therapy provided by PTs, other healthcare professionals delivered additional intervention components such as cognitive therapy, home assessments, family education, and discharge assessments [15,17]. Although the effectiveness of these components was not individually evaluated within each article, it highlights the importance of implementing an interdisciplinary team. This finding is consistent with other systematic reviews in the literature that suggest multi-disciplinary interventions are beneficial when caring for older adults, especially for individuals with CI [28,29]. The results of this review highlight the minimal amount of extant evidence that support health care professionals to provide outpatient rehabilitation interventions for this vulnerable population.

The results of this review indicate that there is a lack of clarity about what community-based rehabilitation interventions for individuals with CI following a hip fracture should involve, and that several substantive gaps require attention to move this field forward. Firstly, only one study described PTs and RNs giving counseling to family members [15]. In the transition from hospital to home, there is a shift of responsibilities to family and other informal caregivers in order to manage the needs of the older adult, thus there is a need for added support and resources for caregivers [27,30]. Future studies should provide emotional and physical support for family caregivers who assume significant roles that are rarely prepared for [31], especially as caregivers become older and may have chronic health issues themselves [32]. Prior to discharge, family members, caregivers, and community care providers are pivotal in translating concepts from an inpatient to an outpatient setting and should be included in discharge planning to increase the consistency of care after discharge. Also, further consideration on how to best leverage and support family caregivers in order to optimize patients’ reintegration to the community, social activities, and other interests outside of the home is needed.

Secondly, there is a need to focus on interventions that are tailored specifically to the patient with CI. Given the debilitating and omnipresent sequela of CI, it would be reasonable to expect that those with CI generally need more individualized care than what standard care currently offers. Since it remains unknown if adapting currently existing frameworks or interventions for those whose cognitive reserve remains intact or using a framework previously developed intervention to include older adults with CI is optimal, perhaps interventions for individuals with CI need to be developed tabula rasa. The needs of older adults with CI may not be addressed by existing rehabilitation programs or standardized checklists intended for a wider, potentially healthier population. More research is required to assess the effectiveness of outpatient rehabilitation programs for older adults with CI following a hip fracture that consist of specific components focused explicitly on physical and cognitive advancements. For example, including a cognitive rehabilitation component that focuses on identifying and addressing individual needs and goals of the patient and targets cognitive functioning, while introducing compensatory methods such as using memory aids [33]. Preliminary results supporting cognitive rehabilitation suggest that more research should be done incorporating such aspects of cognitive rehabilitation with physical rehabilitation in an outpatient setting [33]. In addition, care teams need to involve older adults with CI and their families in care planning to ensure that the care and services are relevant to help them meet their needs. With respect to designing more tailored and individualized interventions, appropriate quality of life measures and a care plan based on the patients’ goals and needs should be integrated and used to comprehensively evaluate intervention success. There is also a need to determine the patient profile that is most suitable for such programs; thus there is a need to include delirium screening and more rigorous cognitive assessments to better understand if the intervention affects different types of CI. Pilot testing of evidence-based interventions using this approach is warranted and the first step to establishing a new framework applicable to this population.

Third, we were unable to compare and evaluate which program components were essential to include in an outpatient rehabilitation program due to the heterogeneity of outcome measures, the lack of description regarding the cognitive function assessments and measures, poor participant description (eg, participants’ comorbidities and baseline data, primary type of CIs), and lack of treatment fidelity monitoring in the included articles. The lack of interventions designed for individuals with CI may be due to a lack of consensus on the proper tools appropriate to measure progress among this population, highlighting the need for increased evidence-based care. Further exploration regarding the corresponding tools that are feasible for the assessment of older adults with CI, and incorporating relevant gold standards for measuring mobility, function, and ability to perform ADLs is warranted. Moreover, greater attention on the comparability of patient performance in a clinical setting versus in the patient’s home would increase our understanding about which measures are best to use. Future research programs should use the same assessment, and measurement tools consistently so that studies can be directly compared to identify what components are most effective for those with CI post hip fracture.

Finally, cost or cost-effectiveness to patient care provision was not an outcome in the included studies which were conducted in Taiwan, Finland, and Australia. The cost of providing hospital care is generally the largest health care cost driver in any health care system, which favors the trend towards co-management models of care [34] or community-based treatments and programs to mitigate care costs [10]. Given the concerns regarding fiscal sustainability in public health care and the general increase in health care spending, future programs that evaluate the economic value of the intervention and include a cost effectiveness analysis are merited.

### Strengths and Limitations

A major strength of this review is that it is comprehensive with the use of a librarian; we used multiple search strategies (electronic search of multiple databases, ancestry search of references) and conducted the search multiple times to ensure the most current evidence was considered. For the electronic search, we searched the databases from inception, and used several terms that are synonymous with community based programs, such as “home-based” and “outpatient,” to ensure the search was inclusive of interventions and programs. We also considered a broad range of outcomes including patient physical function, mobility, and organizational outcomes like emergency room readmissions. As with any review, the findings are constrained by the methodological quality of the included studies. Other reviews [21] considered the evidence in this area to be of “very low quality” with high risk of bias due to the lack of double blinding. However, as in many clinical trials which include the use of health practitioners to deliver the intervention, conducting a double blinded study is challenging and resource intensive which may make it impossible to accomplish in a clinical setting. Lastly, the limitation of including articles published in English and French may have excluded relevant studies conducted in other languages.

### Conclusion

Based on the limited amount of evidence, our review suggests that community-based rehabilitation interventions post hospital discharge from inpatient rehabilitation show promising results to improve physical function outcomes, mobility, and ADLs function 1 year post-discharge from the hospital for older adults with CI, and to increase the likelihood of returning home for a short (3-month) period post-discharge. There is insufficient evidence to indicate the effect of these programs to keep patients at home over the long-term. It currently remains unclear what components an outpatient rehabilitation intervention for individuals with CI following a hip fracture should involve. However, our review findings suggest that interventions should (1) start early in the trajectory of care while the patient is in inpatient rehabilitation and preemptively include discharge planning discussions; (2) be designed with the inclusion of physiotherapy to address the physical component of rehabilitation; and (3) be executed by an interdisciplinary team to provide multifaceted care that continues into the community setting. Given the prevalence of hip fractures in older adults with CI, future research should focus on providing support to the family caregivers as well as including them into the care plan to enhance reintegration into the community, and pilot testing programs that incorporate the goals of the patient and family. A future program of research evaluating these interventions should consider utilizing the same outcome measures, the cognitive function assessments, and detailed participant description (eg, participants’ comorbidities, primary type of CIs) in order to serve as a significant building block towards developing a consistent and expected standard of practice in community-based rehabilitation for older adults with CI following a hip fracture.

## Multimedia Appendix 1

Quality assessment using the Downs and Black checklist.

## Abbreviations

ADLs: activities of daily living
CBI: Chinese Barthel Index
CI: cognitive impairment
MMSE: Mini-Mental State Examination
PPME: Physical Performance and Mobility Exam
PT: physiotherapist
RCT: randomized controlled trial
RN: registered nurse
SPMSQ: Short Portable Mental Status Questionnaire
