# Supplementary material for: Community-Based Hip Fracture Rehabilitation Interventions for Older Adults With Cognitive Impairment: A Systematic Review
Source: JMIR Rehabil Assist Technol. 2016 Jan 22;3(1):e3. doi: 10.2196/rehab.5102 (PMC5454562; doi:10.2196/rehab.5102)
Supplement: Supplementary file 1 [file rehab_v3i1e3_app1.pdf]

Downs And Black checklist questions [14]	Huusko et al [15]	Moseley et al [16]	Shyu et al [17]
1. Is the hypothesis/aim/objective of the study clearly described? Must be explicit<br>Yes=1; No=0	yes=1	yes=1	yes=1
2. Are the main outcomes to be measured clearly described in the Introduction or Methods section? If the main outcomes are first mentioned in the Results section, the question should be answered no. ALL primary outcomes should be described for YES. Yes=1; No=0	no=0	yes=1	yes=1
3. Are the characteristics of the patients included in the study clearly described? In cohort studies and trials, inclusion and/or exclusion criteria should be given. In case-control studies, a case-definition and the source for controls should be given. Single case studies must state source of patient. Yes/No	yes=1	yes=1	yes=1
4. Are the interventions of interest clearly described? Treatments and placebo (where relevant) that are to be compared should be clearly described. Yes/No	yes=1	yes=1	yes=1
5. Are the distributions of principal confounders in each group of subjects to be compared clearly described? A list of principal confounders is provided. YES = age, severity.<br>Yes =2; partially =1; no = 0	yes=2	yes=1	yes=2
6. Are the main findings of the study clearly described? Simple outcome data (including denominators and numerators) should be reported for all major findings so that the reader can check the major analyses and conclusions. Yes/No	yes=1	yes=1	no=0
7. Does the study provide estimates of the random variability in the data for the main outcomes? In non-normally distributed data the inter-quartile range of results should be reported. In normally distributed data the standard error, standard deviation or confidence intervals should be reported. Yes/No	yes=1	yes=1	yes=1
8. Have all important adverse events that may be a consequence of the intervention been reported? This should be answered yes if the study demonstrates that there was a comprehensive attempt to measure adverse events (COMPLICATIONS BUT NOT AN INCREASE IN PAIN).<br>Yes/No	no=0	yes=1	no=0
9. Have the characteristics of patients lost to follow-up been described? If not explicit = NO. RETROSPECTIVE – if not described = UTD; if not explicit re: numbers agreeing to participate = NO. Needs to be >85% Yes=1; No=0	yes=1	No	no=0
10. Have actual probability values been reported (e.g. 0.035 rather than <0.05) for the main outcomes except where the probability value is less than 0.001? Are actual p values listed? Yes=1; No=0	yes=1	yes=1	yes=1
11. Were the subjects asked to participate in the study representative of the entire population from which they were recruited? The study must identify the source population for patients and describe how the patients were selected. Yes=1; No=0; UTD=0	yes=1	yes=1	yes=1
12. Were those subjects who were prepared to participate representative of the entire population from which they were recruited? The proportion of those asked who agreed should be stated. Yes=1; No=0; UTD=0	yes=1	no =160/404	yes=1
13. Were the staff, places, and facilities where the patients were treated, representative of the treatment the majority of patients	yes=1	yes=1	yes=1

receive? For the question to be answered yes the study should demonstrate that the intervention was representative of that in use in the source population. Must state type of hospital and country for YES. Yes=1; No=0; UTD=0			
14. Was an attempt made to blind study subjects to the intervention they have received? For studies where the patients would have no way of knowing which intervention they received, this should be answered yes. Retrospective, single group = NO; UTD if > 1 group and blinding not explicitly stated. Yes=1; No=0; UTD=0	no=0	no=0	no=0
15. Was an attempt made to blind those measuring the main outcomes of the intervention? Must be explicit Yes/No/UTD	no=0	yes=1	no=0
16. If any of the results of the study were based on "data dredging", was this made clear? Any analyses that had not been planned at the outset of the study should be clearly indicated. Retrospective = NO. Prospective. Yes=1; No=0; UTD=0	UTD=0	no=0	yes=1
17. In trials and cohort studies, do the analyses adjust for different lengths of follow-up of patients, or in casecontrol studies, is the time period between the intervention and outcome the same for cases and controls? Where follow-up was the same for all study patients the answer should yes. Studies where differences in follow-up are ignored should be answered no. Acceptable range 1 yr follow up = 1 month each way; 2 years studies, is the time period between the intervention and outcome the same for cases and controls? Yes=1; No=0; UTD=0	yes=1	yes=1	yes=1
18. Were the statistical tests used to assess the main outcomes appropriate? The statistical techniques used must be appropriate to the data. If no tests done, but would have been appropriate to do = NO Yes=1; No=0; UTD=0	yes=1	yes=1	yes=1
19. Was compliance with the intervention/s reliable? Where there was non-compliance with the allocated treatment or where there was contamination of one group, the question should be answered no. Surgical studies will be YES unless procedure not completed. Yes=1; No=0; UTD=0 must be clearly stated if compliance to the intervention was stated	UTD=0	UTD =0	UTD=0
20. Were the main outcome measures used accurate (valid and reliable)? Where outcome measures are clearly described, which refer to other work or that demonstrates the outcome measures are accurate = YES. ALL primary outcomes valid and reliable for YES. Yes/No/UTD	yes=1	yes=1	no=0
21. Were the patients in different intervention groups (trials and cohort studies) or were the cases and controls (case-control studies) recruited from the same population? Patients for all comparison groups should be selected from the same hospital. The question should be answered UTD for cohort and case control studies where there is no information concerning the source of patients Yes/No/UTD	yes=1	yes=1	yes=1
22. Were study subjects in different intervention groups (trials and cohort studies) or were the cases and controls (case-control studies) recruited over the same time? For a study which does not specify the time period over which patients were recruited, the question should be answered as UTD. Surgical studies must be <10 years for YES, if >10 years then NO Yes=1; No=0; UTD=0 date for recruitment time must be clearly stated	UTD=0	yes=1	yes=1
23. Were study subjects randomized to intervention groups? Studies	yes=1	yes=1	yes=1

which state that subjects were randomized should be answered yes except where method of randomization would not ensure random allocation. Yes=1; No=0; UTD=0			
24. Was the randomized intervention assignment concealed from both patients and health care staff until recruitment was complete and irrevocable? All non-randomized studies should be answered no. If assignment was concealed from patients but not from staff, it should be answered no. Yes=1; No=0; UTD=0	no=0	no=0	no=0
25. Was there adequate adjustment for confounding in the analyses from which the main findings were drawn? In non-randomized studies if the effect of the main confounders was not investigated or no adjustment was made in the final analyses the question should be answered as no. If no significant difference between groups shown then YES. Yes/No/UTD	UTD=0	yes=1	yes=1
26. Were losses of patients to follow-up taken into account? If the numbers of patients lost to follow-up are not reported = unable to determine. Yes/No/UTD	yes=1	yes=1	yes=1
27. Did the study have sufficient power to detect a clinically important effect where the probability value for a difference being due to chance <5% Sample sizes have been calculated to detect a difference of x% and y%. 1-5	5	yes=5	0
Total score	23	25	19
